# Heat vortex in hydrodynamic phonon transport of two-dimensional materials

**DOI:** 10.1038/s41598-020-65221-8

**Published:** 2020-05-19

**Authors:** Man-Yu Shang, Chuang Zhang, Zhaoli Guo, Jing-Tao Lü

**Affiliations:** 1 School of Physics and Wuhan National High Magnetic Field Center, Huazhong University of Science and Technology, Wuhan, 430074 P.R. China; 2 State Key Laboratory of Coal Combustion, School of Energy and Power Engineering, Huazhong University of Science and Technology, Wuhan, 430074 P.R. China

**Keywords:** Energy science and technology, Nanoscience and technology, Physics

## Abstract

We study hydrodynamic phonon heat transport in two-dimensional (2D) materials. Starting from the Peierls-Boltzmann equation with the Callaway model approximation, we derive a 2D Guyer-Krumhansl-like equation describing hydrodynamic phonon transport, taking into account the quadratic dispersion of flexural phonons. In addition to Poiseuille flow, second sound propagation, the equation predicts heat current vortices and negative non-local thermal conductance in 2D materials, which are common in classical fluids but have not yet been considered in phonon transport. Our results also illustrate the universal transport behaviors of hydrodynamics, independent of the type of quasi-particles and their microscopic interactions.

## Introduction

Macroscopic collective behavior emerges from microscopic many-body interactions between individual degrees of freedom comprising the system. Hydrodynamics is one of such macroscopic phenomena. It could originate from different kinds of microscopic interactions in different materials, ranging from classical gases and liquids, to crystal solids^1–15^, and to cold atomic gases^16^ or hot nuclear matter^17^. Although the microscopic inter-particle interactions are of different nature, the hydrodynamic behaviors are universal. They can be described by similar hydrodynamic equations. These equations can normally be derived from the microscopic equations of motion by considering physical quantities that are conserved during the inter-particle collisions, i.e., (crystal) momentum, energy or particle number.

Although hydrodynamic flow in classical gases and liquids is a common process that can be observed in everyday life, observing hydrodynamic transport of (quasi-)particles in crystalline solids is much more difficult. Conservation of crystal momentum is required during the inter-particle collisions. This needs high quality samples to reduce extrinsic scatterings with impurities. It also requires that the intrinsic scatterings between quasi-particles are normal (N-process), which conserves the crystal momentum, instead of Umklapp (U-process), which does not. Furthermore, the hydrodynamic features are prominent in spatial confined samples like one-dimensional (1D) or two-dimensional (2D) materials^18–20^, which raises further challenges in their fabrication and characterization.

Due to these limitations, studies on the hydrodynamic transport of quasi-particles in solid state system are scarce. Recently, experimental and numerical signatures of hydrodynamic electron^4–6,12,21–28^ and phonon^8,10,11,29–37^ transport in 2D materials have been reported. For electron transport, negative non-local resistance^4^, violation of Wiedemann-Franz law^5^ and large negative magnetoresistance^21^ have been experimentally observed and theoretically explained^12,22,24–26^. Here non-local thermal conductance/resistance means the temperature difference and the induced heat current (or vice versa) are separated in real space. It can be defined in all the transport regimes, and has been widely used in the literature of electronic transport.

Considering the universal behaviours of hydrodynamics, we expect similar transport behaviours may exist for other quasi-particles in solid. We focus on phonons here. Poiseuille flow and the propagation of second sound have been studied in graphene and similar 2D materials by numerically solving the semi-classical Boltzmann equation with inputs from density functional theory calculation^10,11^. It is suggested that, contrary to three-dimensional materials^2,38–43^, hydrodynamic phonon transport in 2D materials persists over a much larger temperature range(50~150 K) in micrometer scale samples. The quadratic dispersion of graphene ZA acoustic phonon mode is argued to play an important role in widening the temperature range^11^.

However, unlike electrons, experimental evidence of phonon hydrodynamic transport in 2D materials has not been observed, despite recent progress in 3D materials^41,42,44^. Theoretical analysis based on simplified models may help to identify possible experimental signatures of phonon hydrodynamics. Considering its universal behaviors, it is interesting to ask whether similar effects observed for electrons can be expected for phonons. Here, we answer this question from the analysis of a Guyer-Krumhansl (G-K) equation for 2D materials, which we derive from the Peierls-Boltzmann equation with the Callaway model approximation. Importantly, we consider both linear and quadratic acoustic phonon dispersions, which is critical to 2D materials. We extend the multiscale expansion technique^9^ to include both linear and quadratic phonon modes in 2D materials. This has not been considered before. We show that the G-K equation takes a familiar form, but the transport coefficients differ from normal Debye model, which assumes linear dispersion of acoustic phonon modes. The viscosity coefficients, the second sound velocity become temperature dependent, contrary to the Debye model. Our results will be useful for further theoretical and experimental study of phonon hydrodynamics in 2D materials.

## Methods

We consider a prototype 2D material. It has one out-of-plane acoustic mode with a quadratic dispersion ${\omega }_{k}=a{k}^{2}$ (ZA mode), and two degenerate linear acoustic modes ${\omega }_{k}={v}_{g}k$ (longitudinal and transverse). The magnitude of the linear group velocity is *v*_*g*_, and the magnitude of the wave vector is *k* = |***k***|. Here, in the spirit of Debye model, we ignore the possible anisotropic property and the difference between longitudinal and transverse branches. We will focus on the effects of ZA mode with quadratic dispersion on the hydrodynamic behaviors.

We first sketch the derivation of the 2D G-K equation^45,46^. Our starting point is the Peierls-Boltzmann equation under Callaway approximation^47,48^

1 $$\frac{\partial {f}_{s{\boldsymbol{k}}}}{\partial t}+{{\boldsymbol{v}}}_{s{\boldsymbol{k}}}\cdot \nabla {f}_{s{\boldsymbol{k}}}=-\,\frac{{f}_{s{\boldsymbol{k}}}-{f}_{R,s{\boldsymbol{k}}}^{eq}}{{\tau }_{R}}-\frac{{f}_{s{\boldsymbol{k}}}-{f}_{N,s{\boldsymbol{k}}}^{eq}}{{\tau }_{N}}\mathrm{}.$$

Here, *s* is the phonon index, ${\tau }_{N}$ is the constant relaxation time for the N-process, while ${\tau }_{R}$ is that for the resistive scattering process (R-process). It includes all scattering mechanisms that do not conserve crystal momentum, i. e., impurity scattering, electron scattering and other U-processes. We take the same ${\tau }_{R}$ and ${\tau }_{N}$ for all phonon branches, i.e., taking the wave vector and branch averaged values. This is the so-called gray approximation. For quantitative analysis of specific materials, one needs to go beyond the gray approximation and consider the wave vector and branch dependence of $\tau$, which is beyond the scope of present study.

The phonons may reach different steady state distributions due to different kinds of scattering processes, depending on the conserved quantities before and after the scattering. For the N-process, both energy and crystal momentum are conserved. It drives the system towards a displaced Bose-Einstein distribution function

2 $${f}_{N,s{\boldsymbol{k}}}^{eq}={[\exp ({\beta }_{{\rm{B}}}({\rm{\hslash }}{\omega }_{{\rm{s}}{\boldsymbol{k}}}-{\rm{\hslash }}{\boldsymbol{k}}{\rm{\cdot }}{\boldsymbol{u}}))-1]}^{-1},$$

with ${\beta }_{B}={({k}_{{\rm{B}}}T)}^{-1}$, and ***u*** is the drift velocity. But for the R-process, only energy is conserved. It drives the system to the equilibrium Bose-Einstein distribution

3 $${f}_{R,s{\boldsymbol{k}}}^{eq}={(\exp ({\beta }_{{\rm{B}}}{\rm{\hslash }}{\omega }_{{\rm{sk}}})-1)}^{-1}.$$

It has been shown numerically that, within some moderate temperature range (~100 K for graphene), the N-process is orders of magnitude faster than the R-process, meaning ${\tau }_{R}\gg {\tau }_{N}$ ^10,11^. When the system size is much larger than the normal-scattering mean free path $l\approx {v}_{g}{\tau }_{N}$. The relaxation from local to global equilibrium or steady state is governed by hydrodynamic equations describing the conserved quantities during the *N*-process, including energy and crystal momentum.

As we know, conservation laws play central roles in the derivation of hydrodynamic equations. Since phonons can be generated or destroyed during the scattering processes, the number of phonons is not conserved. We consider energy and crystal momentum conservation here. Both normal (N) and resistive (R) scattering processes conserve total energy, giving

4 $$\sum _{s}\int \frac{d{\boldsymbol{k}}}{{\mathrm{(2}\pi )}^{2}}\hslash {\omega }_{{\rm{s}}{\boldsymbol{k}}}{f}_{{\rm{s}}{\boldsymbol{k}}}=\sum _{s}\int \frac{d{\boldsymbol{k}}}{{\mathrm{(2}\pi )}^{2}}\hslash {\omega }_{{\rm{s}}{\boldsymbol{k}}}{\,f}_{N,s{\boldsymbol{k}}}^{eq},$$

5 $$\sum _{s}\int \frac{d{\boldsymbol{k}}}{{\mathrm{(2}\pi )}^{2}}\hslash {\omega }_{s{\boldsymbol{k}}}{f}_{s{\boldsymbol{k}}}=\sum _{s}\int \frac{d{\boldsymbol{k}}}{{\mathrm{(2}\pi )}^{2}}\hslash {\omega }_{s{\boldsymbol{k}}}{\,f}_{R,s{\boldsymbol{k}}}^{eq},$$

while only the N-process obeys crystal momentum conservation, giving

6 $$\sum _{s}\int \frac{d{\boldsymbol{k}}}{{\mathrm{(2}\pi )}^{2}}\hslash {\boldsymbol{k}}{f}_{s{\boldsymbol{k}}}=\sum _{s}\int \frac{d{\boldsymbol{k}}}{{\mathrm{(2}\pi )}^{2}}\hslash {\boldsymbol{k}}{f}_{N,s{\boldsymbol{k}}}^{eq}\mathrm{}.$$

### Energy balance equation

To get the equation governing the dynamics of the conserved quantity, we multiply by $\hslash {\omega }_{s{\bf{k}}}$, integrate over ***k*** and sum over the phonon index on both sides of Eq. (1). We then arrive at an equation describing energy conservation

7 $$\frac{\partial E}{\partial t}+\nabla \cdot {\boldsymbol{q}}=\mathrm{0,}$$

where

8 $$E=\sum _{s}\int \frac{d{\boldsymbol{k}}}{{\mathrm{(2}\pi )}^{2}}\hslash {\omega }_{s{\boldsymbol{k}}}{f}_{s{\boldsymbol{k}}}$$

is the energy density, and

9 $${\boldsymbol{q}}=\sum _{s}\int \frac{d{\boldsymbol{k}}}{{\mathrm{(2}\pi )}^{2}}\hslash {\omega }_{s{\boldsymbol{k}}}{{\boldsymbol{v}}}_{s{\boldsymbol{k}}}{f}_{s{\boldsymbol{k}}}$$

is the heat flux. The right hand side of Eq. (7) is zero because the scattering processes conserve energy.

### Momentum balance equation

Multiplying $\hslash {\boldsymbol{k}}$ and performing the integration/summation, we can obtain an equation for the crystal momentum density ***p*** and its flux $\overleftrightarrow{{\boldsymbol{\Phi }}}$ from its conservation law during *N*-process

10 $$\frac{\partial {\boldsymbol{p}}}{\partial t}+\nabla \cdot \overleftrightarrow{{\boldsymbol{\Phi }}}=-\,\frac{{\boldsymbol{p}}}{{\tau }_{R}},$$

with the momentum density

11 $${\boldsymbol{p}}=\sum _{s}\int \frac{d{\boldsymbol{k}}}{{\mathrm{(2}\pi )}^{2}}\hslash {\boldsymbol{k}}{f}_{s{\boldsymbol{k}}}\,,$$

and momentum flux tensor

12 $$\overleftrightarrow{{\boldsymbol{\Phi }}}=\sum _{s}\int \frac{d{\boldsymbol{k}}}{{\mathrm{(2}\pi )}^{2}}{{\boldsymbol{v}}}_{s{\boldsymbol{k}}}\hslash {\boldsymbol{k}}{f}_{s{\boldsymbol{k}}}\mathrm{}\,.$$

### The heat-flux equation

We can also write down a heat-flux equation by multiplying $\hslash {\omega }_{s{\boldsymbol{k}}}{{\boldsymbol{v}}}_{s{\boldsymbol{k}}}$ to each term in Eq. (1) and performing similar summation. The resulting equation reads

13 $$\frac{\partial {\boldsymbol{q}}}{\partial t}+\frac{\overleftrightarrow{{\boldsymbol{\kappa }}}}{{\tau }_{R}}\cdot \nabla T=-\,\frac{{\boldsymbol{q}}}{{\tau }_{R}}-\frac{{\boldsymbol{q}}-{{\boldsymbol{q}}}_{0}}{{\tau }_{N}}\mathrm{}.$$

Here, the presence of last term at the right side of the equation is due to the fact that normal scattering process conserves crystal momentum, but not the heat flux

14 $${\boldsymbol{q}}=\sum _{s}\int \frac{d{\boldsymbol{k}}}{{\mathrm{(2}\pi )}^{2}}\hslash {\omega }_{s{\boldsymbol{k}}}{{\boldsymbol{v}}}_{s{\boldsymbol{k}}}{f}_{s{\boldsymbol{k}}}\mathrm{}.$$

Here, ***q***_0_ is defined similarly by replacing *f*_*s****k***_ by ${f}_{N,s{\boldsymbol{k}}}^{eq}$. We have defined the thermal conductivity tensor

15 $$\overleftrightarrow{{\boldsymbol{\kappa }}}={\tau }_{R}\sum _{s}\int \frac{d{\boldsymbol{k}}}{{\mathrm{(2}\pi )}^{2}}\hslash {\omega }_{s{\boldsymbol{k}}}{{\boldsymbol{v}}}_{s{\boldsymbol{k}}}{{\boldsymbol{v}}}_{s{\boldsymbol{k}}}\frac{\partial {f}_{s{\boldsymbol{k}}}}{\partial T}\mathrm{}.$$

### Multi-scale expansion

To obtain the hydrodynamic heat transport equation, we follow a multi-scale expansion technique^9^ and extend it to the case of 2D material with one quadratic phonon dispersion. This quadratic phonon dispersion is important in 2D materials like graphene^11^. The expansion is over both space and time as follows:

16 $$\frac{\partial }{\partial {x}_{i}}=\varepsilon \frac{\partial }{\partial {x}_{1i}},$$

17 $$\frac{\partial }{\partial t}=\varepsilon \frac{\partial }{\partial {t}_{1}}+{\varepsilon }^{2}\frac{\partial }{\partial {t}_{2}}\mathrm{}.$$

It is a perturbation expansion over a natural small parameter

18 $$\varepsilon =\frac{{\tau }_{N}}{{\tau }_{R}}\mathrm{}.$$

This means we consider the situation where the scattering rates of N-process are much larger than that of R-process. This is the necessary condition for hydrodynamic phonon transport. The phonon distribution function *f* can be expanded similarly

19 $$f=\sum _{n}{\varepsilon }^{n}{f}_{n},$$

with the *n*-th order distribution *f*_*n*_. The macroscopic variables can be expressed by the sum of corresponding components. For the energy density, we have

20 $$E=\sum _{n}{\varepsilon }^{n}{E}_{n},\,{E}_{n}=\sum _{s}\int \hslash {\omega }_{s{\boldsymbol{k}}}{f}_{n,s{\boldsymbol{k}}}\frac{d{\boldsymbol{k}}}{{\mathrm{(2}\pi )}^{2}}\mathrm{}.$$

Other quantities are defined similarly.

According to energy and momentum conservation (Eqs. (4) and (6)), we know that *E* = *E*_0_, ***p*** = ***p***_0_. If all the phonon modes follow linear dispersion, ***q*** is simplied proportional to ***p*** (Eq. (30)), thus ***q*** = ***q***_0_. This is the case for the Debye model. However, for 2D materials, when we include the quadratic phonon mode, the simple relation does not hold any more, ***q*** ≠ ***q***_0_.

All the defined quantities are calculated from the distribution function

21 $$f\approx {f}_{0}+\varepsilon {f}_{1},$$

with

22 $${f}_{0}={f}_{N}^{eq},$$

23 $${f}_{1}={f}_{R}^{eq}-{f}_{N}^{eq}-{\tau }_{N}({\partial }_{{t}_{1}}\,{f}_{N}^{eq}+{v}_{i}{\partial }_{{x}_{1i}}\,{f}_{N}^{eq})\mathrm{}.$$

In most cases, we can approximate ${f}_{N}^{eq}$ as

24 $${f}_{N}^{eq}\approx {f}_{R}^{eq}+\hslash {\beta }_{B}\,{f}_{R}^{eq}(\,{f}_{R}^{eq}+\mathrm{1)}{\boldsymbol{k}}\cdot {\boldsymbol{u}}.$$

Taking into account only the 0th order term *f*_0_, we can get

25 $${E}_{L}={E}_{0L}=\frac{2Z\mathrm{(3)}}{\pi }\frac{{({k}_{B}T)}^{3}}{{(\hslash {v}_{g})}^{2}},$$

26 $${E}_{N}={E}_{0N}=\frac{\pi }{24}\frac{{({k}_{B}T)}^{2}}{\hslash a},$$

27 $${{\boldsymbol{q}}}_{0L}=\frac{3}{2}{E}_{L}{\boldsymbol{u}},\,{{\boldsymbol{q}}}_{0N}=2{E}_{N}{\boldsymbol{u}},$$

28 $${\overleftrightarrow{{\boldsymbol{\kappa }}}}_{0L}=\frac{1}{2}{C}_{L}{v}_{g}^{2}{\tau }_{R}\overleftrightarrow{{\boldsymbol{I}}},\,{\overleftrightarrow{{\boldsymbol{\kappa }}}}_{0N}={\overleftrightarrow{{\boldsymbol{\kappa }}}}_{0L},$$

29 $${\overleftrightarrow{{\boldsymbol{\Phi }}}}_{0L}=\frac{1}{2}{E}_{L}\overleftrightarrow{{\boldsymbol{I}}},\,{\overleftrightarrow{{\boldsymbol{\Phi }}}}_{0N}={E}_{N}\overleftrightarrow{{\boldsymbol{I}}},$$

30 $${{\boldsymbol{p}}}_{L}={{\boldsymbol{p}}}_{0L}=\frac{3}{2}\frac{{E}_{L}}{{v}_{g}^{2}}{\boldsymbol{u}}=\frac{1}{{v}_{g}^{2}}{{\boldsymbol{q}}}_{0L}\mathrm{}.$$

Here, *Z*(*x*) is the Riemann Zeta function, the index *L* and *N* represent the linear and quadratic phonon contributions, respectively.

The calculation of ***p***_0*N*_ needs special care. Simple calculation using Eq. (24) leads to divergent result. We need to use the full form of ${f}_{N}^{eq}$ instead of Eq. (24) and obtain

31 $${{\boldsymbol{p}}}_{N}={{\boldsymbol{p}}}_{0N}=\frac{{k}_{B}T}{8\pi {a}^{2}}\left[-\,\mathrm{ln}\left(1-\exp \frac{\hslash {u}^{2}}{4{k}_{B}Ta}\right)\right]{\boldsymbol{u}}\mathrm{}.$$

We see that it depends on ***u*** non-linearly, and the linear coefficient diverges as $u\to 0$. As a result, we have to consider a finite ***u*** when calculating ***p***_*N*_. This makes the linear approximation in ***u*** and consequently the derivation of G-K equation using the momentum balance equation difficult. The other complication that the quadratic mode brings is the lack of simple proportionality between ***p***_*N*_ and ***q***_*N*_. For linear dispersion, we have ${{\boldsymbol{q}}}_{0L}={v}_{g}^{2}{{\boldsymbol{p}}}_{0L}$. As a result, when only considering phonons with linear dispersion, the same G-K equation can be derived starting from either the momentum balance or the heat-flux equation. The inclusion of the quadratic dispersion leads to two different G-K-like equations, describing momentum and heat flow, respectively. Here, we focus on the heat transport, and proceed from the heat-flux equation (13). We get the first order correction to the thermal conductivity

32 $${\overleftrightarrow{{\boldsymbol{k}}}}_{1}={\tau }_{R}\frac{\partial \overleftrightarrow{{\boldsymbol{Q}}}}{\partial T}={\tau }_{R}(\nabla \cdot \overleftrightarrow{{\boldsymbol{Q}}})\frac{1}{\nabla T}\mathrm{}.$$

The expression of $\overleftrightarrow{{\boldsymbol{Q}}}$ and the details of the derivation can be found in the Supplementary Information (SI).

### The 2D G-K equation

Substituting the above results into Eq. (13), and neglecting ***q***_1_, we arrive at the G-K-like equation for heat transport

33 $$\frac{\partial {\boldsymbol{q}}}{\partial t}+\frac{{\kappa }_{0}}{{\tau }_{R}}\nabla T+\frac{1}{{\tau }_{R}}{\boldsymbol{q}}=\eta [{\nabla }^{2}{\boldsymbol{q}}+2\nabla (\nabla \cdot {\boldsymbol{q}})]-\zeta \nabla (\nabla \cdot {\boldsymbol{q}}\mathrm{)}.$$

Here, considering the isotropic case, the transport coefficients can be expressed as numbers. ${\kappa }_{0}$ is the magnitude of the zeroth order thermal conductivity, $\eta$ and $\zeta$ are the first, second viscosity coefficients, respectively. All the three transport coefficients can be divided into contributions from the linear and quadratic phonon modes, respectively. Denoting them with subscripts *L* and *N*, we have ${\kappa }_{0L}={\kappa }_{0N}={C}_{L}{v}_{g}^{2}{\tau }_{R}\mathrm{/2}$, ${\eta }_{L}={\eta }_{N}\mathrm{/2}=3{E}_{L}\mathrm{/(8}\bar{E}){v}_{g}^{2}{\tau }_{N}$, ${\zeta }_{L}={\zeta }_{N}={C}_{L}\mathrm{/(2}C){v}_{g}^{2}{\tau }_{N}$. It can be checked that, our results reduce to that of Debye model if we ignore the quadratic phonon mode. We can also write ${\kappa }_{0}=\alpha C{v}_{g}^{2}{\tau }_{R}$, $\eta =\beta {v}_{g}^{2}{\tau }_{N}$, $\zeta =\alpha {v}_{g}^{2}{\tau }_{N}$, respectively, where $\alpha ={C}_{L}/C$, $\beta =9{E}_{L}\mathrm{/(8}\bar{E})$, with $\bar{E}=\frac{3}{2}{E}_{L}+2{E}_{N}$. Equation (33) with the above defined coefficients is the central result of this work. We can see that, the form of the G-K equation is the same as the 3D case. The inclusion of quadratic phonon mode changes its coefficients. Notably, $\eta$ and $\zeta$ become temperature dependent, while for Debye model with three degenerate linear acoustic phonons modes, the coefficients are constant, with $\alpha =\mathrm{1/2}$, $\beta =\mathrm{1/4}$ for 2D case (obtained by setting ${\kappa }_{0N}={\eta }_{N}={\zeta }_{N}\mathrm{=0}$) and $\alpha =\mathrm{1/3}$, $\beta =\mathrm{1/5}$ for 3D^9^, respectively.

We note by passing that, in deriving Eq. (33) we have made the approximation ${\boldsymbol{q}}\approx {{\boldsymbol{q}}}_{0}$. Inclusion of ***q***_1_ requires solution of higher order equations in the expansion over $\varepsilon$. Analytical treatment becomes difficult, if not impossible. Thus, we rely on fully numerical solution of the Callaway model to check its validity, as we have done in Fig. 3. We show that our G-K equation can reasonably re-produce the main features of the hydrodynamic heat flow that we focus in this work. Physically, this approximation should hold when $\varepsilon \ll 1$ or ${\tau }_{N}\ll {\tau }_{R}$, which is the parameter range we consider here. One additional support for our approximation is that the viscosity coefficients, which come from ${\overleftrightarrow{{\boldsymbol{\kappa }}}}_{1}$, do not depend on ***q***_1_ (see Sec. 2.2 of SI).

**Figure 1:**
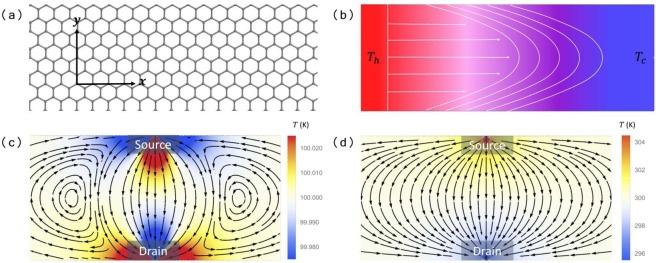
(**a**) A graphene nano-ribbon as a prototype 2D materials showing hydrodynamics phonon transport. (**b**) Schematic of Poiseuille flow generated by the temperature difference along the nano-ribbon. The heat flux has a parabolic distribution across the ribbon. (**c**) Heat current loops (lines) and temperature distribution (color) due to the viscosity of the phonon gas as a signature of hydrodynamic heat transport. We set $\chi =0.5$, and the average temperature $\bar{T}=100$ K. (**d**) The same as (**c**), but in the diffusive Fourier transport regime with $\chi =2\times {10}^{4}$ and $\bar{T}=300$ K.

**Figure 2:**
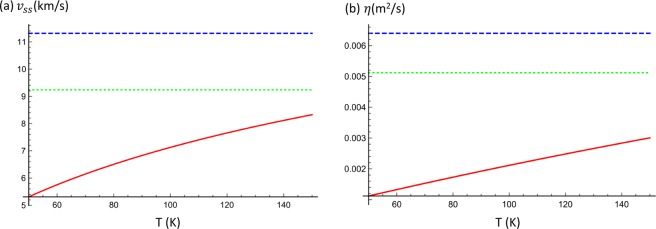
(**a**) The second sound velocity *v*_*ss*_ as a function of temperature (T) for three different situations. (**b**) The dependence of the first viscosity coefficient $\eta$ on temperature (T). Here, we take the ${\tau }_{N}={10}^{-10}$ s. The green dotted line corresponds to the 3D Debye model with group velocity ${v}_{g}=1.6\times {10}^{4}$ m/s, the blue dashed line is the 2D Debye model with the same group velocity, while the red solid line stands for the 2D case with one quadratic ZA mode and two degenerate linear acoustic modes (longitudinal and transverse) with the same *v*_*g*_.

**Figure 3:**
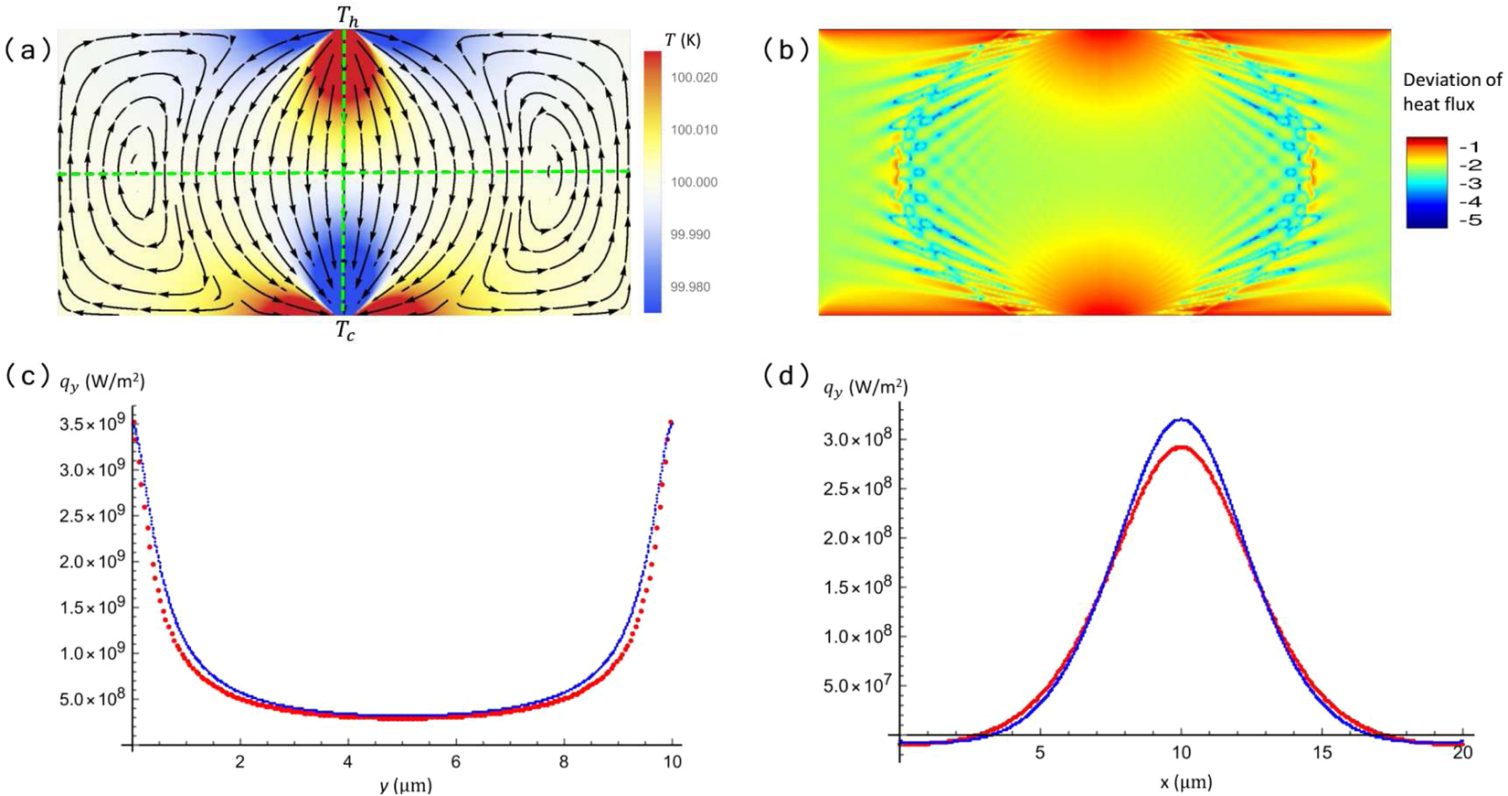
(**a**) Numerical results obtained by solving the Peierls-Boltzmann equation under the Callaway approximation using the discrete ordinate method. A temperature difference is applied such that the heat flux injected into the ribbon is the same as Fig. 1(c), ${\tau }_{R}={10}^{-7}$ s, ${\tau }_{N}={10}^{-10}$ s, $\chi =0.5$ the same as that in Fig. 1(c). This is compared to Fig. 1(c). The differences of heat current streamlines near the left and right boundaries are due to the periodic boundary condition used in the numerical calculation. (**b**) Numerical results for the spatial distribution of heat flux deviation, plotted in logarithmic scale $\mathrm{lg}|({\boldsymbol{q}}-{{\boldsymbol{q}}}_{0})/{\boldsymbol{q}}|$. The deviation is fairly small in general, especially around the vortices. The maximum deviation locates at the upper and lower boundaries due to the *δ*-distributed source and drain current. (**c,d**) Heat flux in *y* direction (*q*_*y*_) along the vertical (**c**) and horizontal (**d**) line cuts shown in (**a**) (green dashed lines). The theoretical results taken from Fig. 1(c) are shown in blue, and the numerical ones taken from (**a**) are shown in red. The equilibrium layer distance *d* = 3.35 Å is used to convert *q*_*y*_ into the standard unit. The coordinate of the ribbon center is (*x* = 10, *y* = 5) *μ*m.

## Results

### Second sound

The right side (RHS) of the G-K equation represents the effect of viscosity on the heat transport behavior. They come from the first order term in the expansion over $\varepsilon$. Before looking into these terms, we show here that the propagation of second sound can be analyzed without these terms. Replacing the RHS with zero, combining with Eq. (7), we arrive at

34 $$\frac{{\partial }^{2}T}{\partial {t}^{2}}+\frac{1}{{\tau }_{R}}\frac{\partial T}{\partial t}-{v}_{ss}^{2}\sum _{i=x,y,z}\frac{{\partial }^{2}T}{\partial {r}_{i}^{2}}=0,$$

where we have defined the second sound velocity

35 $${v}_{ss}^{2}=\frac{{\kappa }_{0}}{C{\tau }_{R}}=\alpha {v}_{g}^{2}\mathrm{}.$$

This is the wave equation describing propagation of second sound with velocity *v*_*ss*_ and damping coefficient ${\tau }_{R}^{-1}$. For 3D materials with the Debye model, the second sound velocity is ${v}_{ss}={v}_{g}/\sqrt{3}$, similar model for 2D material gives ${v}_{ss}={v}_{g}/\sqrt{2}$. Here, the presence of quadratic dispersion makes *v*_*ss*_ temperature dependent, inherited from the different temperature dependence of *C*_*L*_ and *C*_*N*_ [see Fig. 2(a)]. We find that, in the presence of quadratic ZA modes, *v*_*ss*_ increases with temperature and is much smaller than results from the Debye model in the relevant temperature ~100 K. The physical mechanism is the following. The quadratic dispersion of ZA modes have frequency dependent phonon group velocity, i.e., ${v}_{g}\propto k$, and *k*-independent constant density of states. This is contrast to constant *v*_*g*_ and linear-in-*k* density of states of phonons with linear dispersion (LA and TA modes). At low temperature, phonons with small *k* contribute dominantly to the propagation of second sound. Since ZA modes contribute dominantly, *v*_*ss*_ is much smaller than the constant *v*_*g*_ of linear phonons. When the temperature increases, more linear phonons contribute and *v*_*ss*_ increases correspondingly.

### Poiseuille flow

We now include the RHS of the G-K equation, and consider a nano-ribbon with length *L* ($0\le x\le L$) and width *w* ($0\le y\le w$) [Fig. 1(a)]. A temperature difference is applied along the ribbon (*x* direction) [Fig. 1(b)]. At steady state, ignoring ${\boldsymbol{q}}/{\tau }_{R}$, Eq. (33) reduces to a one-dimension form ${\partial }^{2}q/\partial {y}^{2}=A$. This gives rise to a parabolic heat flux distribution perpendicular to the flow $q(y)=Ay(y-w\mathrm{)/2}$, with $A=(\partial T/\partial x){\kappa }_{0}/{\tau }_{R}\eta$, if we assume a non-slip boundary condition $q\mathrm{(0)}=q(w)=0$. By integration over *y*, the heat current is obtained

36 $$I={\int }_{0}^{w}q(y)dy=-\,\frac{1}{12}A{w}^{3}\mathrm{}.$$

The negative sign means heat flows opposite to the temperature gradient. The heat current scaling as *w*^3^ is a signature of the Poiseuille flow. For diffusive phonon transport, the heat current scales linearly with the ribbon width $I\propto w$, while for ballistic transport, the heat current can not go higher than linear scaling with the width. Thus, the cubic (super-linear) dependence of *I* on *w* can in principle be used as a signature of the Poiseuille flow. The Poiseuille flow in graphene ribbons has been studied numerically by solving the Boltzmann equation directly in refs. ^10,11^. Similar behavior is also predicted for electronic transport in graphene nano-ribbons^12^. Length and width dependent thermal conductivity in suspended single layer graphene has been reported experimentally^49,50^. Thus, experimental confirmation of Poiseuille flow in graphene is already within reach.

### Steady state heat flow in a ribbon

One important consequence of Eq. (33) is the formation of heat flow vortices when there is a heat current source injecting into the 2D materials. As far as we know, this has not been considered before, on which we focus in this work. We consider steady state transport in a setup sketched in Fig. 1(c). Heat current source and drain are attached to a graphene nano-ribbon. The pattern of heat current flow at steady state can be obtained from the solution of simplified version of Eq. (33). At steady state, according to Eq. (7), we have $\partial {\boldsymbol{q}}/\partial t=\partial E/\partial t=0$. The resulting equation has the form

37 $$\eta {\nabla }^{2}{\boldsymbol{q}}-{\tau }_{R}^{-1}{\boldsymbol{q}}={C}_{L}{v}_{g}^{2}\nabla T\mathrm{}.$$

It shares the same form as the electronic case in ref. ^12^. The 1st term on the left hand side (LHS) is the hydrodynamic term due to viscosity. When ${\tau }_{R}^{-1}$ is negligible, we get a pure hydrodynamic viscous flow. On the other hand, when $\eta$ is negligible, we recover the normal diffusive heat transport governed by Fourier law. Actually, it is suggestive to define a dimensionless parameter

38 $$\chi =\frac{{w}^{2}}{\eta {\tau }_{R}}=\frac{{w}^{2}}{\beta {v}_{g}^{2}{\tau }_{N}{\tau }_{R}}$$

to characterize the relative contribution of diffusive and hydrodynamic transport. *χ* basically characterizes the relative contribution of the first and the second term in Eq. (37). In the limit of $\chi \to +\,\infty$, the R-process is dominant, Eq. (37) reduces to the diffusive Fourier law. However, in the other limit $\chi \to 0$, the viscosity term dominates.

Following ref. ^12^, we have solved Eq. (37) analytically with the help of the streaming function (see Sec. 3 of the SI for details). As an example, we have plotted typical heat current flow patterns (lines) and the resulting temperature distribution (color) with $\chi =0.5$ and 2 × 10^4^ in Fig. 1(c,d), respectively. Here, a flow of heat current from a point source $I(x)=I\delta (x)$ is injected into the ribbon and collected at the opposite side. The non-slip boundary condition is used to solve Eq. (37). The heat current flow within the ribbon can be obtained from the solutions. For small *χ* or larger $\eta$ [Fig. 1(c)], hydrodynamic transport is dominant. The formation of vortices at both sides of the direct source-to-drain flow is a characteristic feature of the viscous flow. This feature is shared by different kinds of classical or quantum fluid. Similar behavior of electrons in graphene and other 2D materials has received intense research focus very recently^4–6,9,22,51^. As a results of vortices formation, there appears the separation of temperature gradient and heat flow. Even negative thermal resistance can be observed, where the heat current flows are from the low to the high temperature regime. This is an obvious violation of the Fourier law. For larger *χ* [Fig. 1(d)], the system is in the diffusive Fourier transport regime. Heat current vortices and negative thermal resistance are absent. Thus, we can realize a transition from hydrodynamic to Fourier transport by changing the magnitude of *χ*.

Here, it is worth mentioning that, for much smaller nanoscale systems, the wave property of phonons and complicated elastic boundary scattering may also leads to the formation of vortices in the ballistic transport regime^34^. Despite the similarity, the physical mechanism and length scale are quite different from the viscous flow studied here. In the ballistic case, coherent phonon transport together with elastic boundary scattering is the physical mechanism to generate heat vortices. Here in the hydrodynamic case, vortex formation is due to frequent momentum-conserving N-processes together with the boundary conditions imposed here, i.e., heat current injection and collection at local positions. It is a result of frequent momentum exchange between different phonons, akin to the vortex formation in classical gas or liquid flow. While the ballistic phonon transport takes place in nanoscale ribbons, the hydrodynamic transport takes place in the microscale (See below for the estimation of length scale in graphene).

As we mentioned above, in deriving Eq. (33), we have considered only the zeroth order term of the heat current ***q***. To check the validity of this truncation and the results plotted in Fig. 1(c), we have performed fully numerical calculation by solving the Boltzmann equation under Callaway approximation using the discrete ordinate method^33,52^. More details of the numerical calculation can be found in refs. ^33,52^. We consider the same ribbon at the same average temperature (${T}_{0}=100$ K) as in Fig. 1(c), with the same dimensionless parameter *χ*. The temperature difference in the numerical calculation is chosen such that the heat flux ***q*** flowing into the ribbon is the same as that in Fig. 1(c). In this way, we can compare directly the results obtained from the two methods. The numerical calculation serves as a benchmark for our truncation ***q***_1_ = 0. The results are plotted in Fig. 3(a). Comparing with Fig. 1(c), we can find that the main features of hydrodynamic heat flow are re-produced by the G-K heat equation derived here. Figure 3(b) shows the distribution of relative difference between ***q*** and ***q***_0_ both obtained from the numerical calculation. We have checked that, the maximum relative difference, defined as |(***q*** − ***q***_0_)/***q***|, locates at the upper and lower boundaries. Part of the difference comes from the singular boundary conditions, i.e., the heat current injected and collected are distributed as delta changes. The relative difference is rather small away from the two boundaries, especially around the vortices. As examples, we have plotted the distribution of heat flux along two line cuts (green dashed lines in Fig. 3) passing the center of the ribbon in *y* (Fig. 3(c)) and *x* (Fig. 3(d)) directions, respectively. The analytical and numerical results show good agreements in both cases. This further validates our study of the hydrodynamic heat flow using the G-K equation derived here.

### Application to graphene

We now give an order-of-magnitude analysis using parameters of graphene. We obtain the phonon dispersion relation of graphene using density function theory based calculations (For the density functional theory calculation, we use the Vienna Ab-initio Simulation Package and the generalized gradient approximation for the exchange-correlation functional. The parameters are the same as ref. ^53^). Fitting the dispersion relation results in ${v}_{g}=1.6\times {10}^{4}$ m/s for the linear modes, $a=5.5\times {10}^{-7}$ m^2^/s for the quadratic mode. The specific heat capacity of them are given by ${C}_{L}=\frac{6Z\mathrm{(3)}}{\pi }\frac{{k}_{B}^{3}{T}^{2}}{{(\hslash {v}_{g})}^{2}}\approx 2.14\times {10}^{-9}{T}^{2}$ Jm^−2^ K^−1^ and ${C}_{N}=\frac{\pi }{12}\frac{{k}_{B}^{2}T}{\hslash a}\approx 8.63\times {10}^{-7}T$ Jm^−2^ K^−1^, respectively. To estimate the transport coefficients and the dimensionless factor *χ*, we use ${\tau }_{N}={10}^{-10}$ s, ${\tau }_{R}={10}^{-7}$ s^11^. We get $\beta \approx 0.08$ at $T=100$ K, smaller than value obtained from the 2D Debye model $\beta =\mathrm{1/4}$. In contrast to 2D or 3D Debye model, the different temperature dependences of *C*_*L*_, *C*_*N*_ and *E*_*L*_, *E*_*N*_ give rise to temperature dependent $\eta$ (Fig. 2(b)). We get $\eta \approx 0.002$ m^2^/s at $T=100$ K, which is orders of magnitude larger than that of water. We also get the dimensionless parameter $\chi \approx 5\times {10}^{9}{w}^{2}$ m^−2^. Thus, phonon hydrodynamic transport can be realized in high quality graphene nano-ribbons of micrometer scale. The plot in Fig. 1(c) with $\chi =0.5$ corresponds to sample size of ~10 *μ*m.

## Conclusions

In summary, we have derived a 2D version of the G-K equation describing hydrodynamic phonon heat transport. We take into account the out of plane quadratic phonon dispersion of the ZA mode, normally present in 2D materials. Its effect on the hydrodynamic transport is analyzed. The derived equation serves as a starting point for investigating hydrodynamic phonon transport behavior in 2D materials. It shares a similar form as the Navier-Stokes equation that has been used to study electron hydrodynamic transport in graphene. Many interesting transport behaviors, including non-local negative resistance, higher-than-ballistic transport, predicted for electrons can be studied for phonons. Moreover, a large overlap of the parameter regime between electron and phonon hydrodynamic transport in graphene makes it promising to study the effect of their mutual interaction on the thermal transport behavior of the two kinds of fluids.

## Supplementary information

Supplementary information.
