# Supplementary material for: Heat vortex in hydrodynamic phonon transport of two-dimensional materials
Source: Sci Rep. 2020 May 19;10:8272. doi: 10.1038/s41598-020-65221-8 (PMC7237709; doi:10.1038/s41598-020-65221-8)
Supplement: Supplementary file 1 — Supplementary information. [file 41598_2020_65221_MOESM1_ESM.pdf]

Supplementary Information for Heat vortex in hydrodynamic phonon transport of two-dimensional materials

Man-Yu Shang[1], Chuang Zhang[2], Zhaoli Guo[2], and Jing-Tao Lü[1]

[1]School of Physics and Wuhan National High Magnetic Field Center, Huazhong University of Science and Technology, Wuhan 430074, P. R. China
[2]State Key Laboratory of Coal Combustion, School of Energy and Power Engineering, Huazhong University of Science and Technology, Wuhan 430074, P. R. China

1 The steady state distribution functions

The steady state distribution functions are obtained by considering the conserved quantities during the scattering process. For N-process, both crystal momentum and energy are conserved. If we take a 3-phonon scattering process as an example, we have

$$\omega_{\boldsymbol{k}_1} + \omega_{\boldsymbol{k}_2} = \omega_{\boldsymbol{k}_3}, \tag{S1}$$

$$\boldsymbol{k}_1 + \boldsymbol{k}_2 = \boldsymbol{k}_3. \tag{S2}$$

On the other hand, when reaching the steady state, the forward and backward scattering processes are balanced. Thus, the phonon distribution function satisfies

$$f_{\boldsymbol{k}_1} f_{\boldsymbol{k}_2}(1 + f_{\boldsymbol{k}_3}) = (1 + f_{\boldsymbol{k}_1})(1 + f_{\boldsymbol{k}_2}) f_{\boldsymbol{k}_3}. \tag{S3}$$

We can write the above equation in a different form

$$\ln(1 + f_{\boldsymbol{k}_1}^{-1}) + \ln(1 + f_{\boldsymbol{k}_2}^{-1}) = \ln(1 + f_{\boldsymbol{k}_3}^{-1}). \tag{S4}$$

Comparing Eqs. (S1-S4), we find that we can write the distribution function in terms of the conserved quantities

$$\ln(1 + f_{\boldsymbol{k}}^{-1}) = \beta(\hbar\omega - \hbar\boldsymbol{k}\cdot\boldsymbol{u}). \tag{S5}$$

This results in a displaced Bose-Einstein distribution function

$$f_{\boldsymbol{k}} = \frac{1}{e^{\beta(\hbar\omega - \hbar\boldsymbol{k}\cdot\boldsymbol{u})} - 1}. \tag{S6}$$

Similarly, we can show that the U-process drives the phonons to the equilibrium Bose-Einstein distribution.

2 Derivation of the 2D G-K equation

Here, we give the derivation of 2D G-K equation in the main text. We use the recent developed multiscale expansion technique[?]. The expansion is over both space and time as follows:

$$\frac{\partial}{\partial x_i} = \varepsilon \frac{\partial}{\partial x_{1i}}, \tag{S7}$$

$$\frac{\partial}{\partial t} = \varepsilon \frac{\partial}{\partial t_1} + \varepsilon^2 \frac{\partial}{\partial t_2}, \tag{S8}$$

where ε is a small parameter, defined as

$$\varepsilon = \frac{\tau_N}{\tau_R}. \tag{S9}$$

This means that we consider the situation where the scattering rates of N-process are much larger than that of R-process. This is the necessary condition for hydrodynamic phonon transport. The phonon distribution function f can be expanded similarly

$$f = f_0 + \varepsilon f_1 + \varepsilon^2 f_2 + \cdots, \tag{S10}$$

with the 0th, 1st and 2nd order distribution function f_0, f_1, f_2. The macroscopic variables can be expressed by the sum of corresponding components. Substituting all of the equations above into the Peierls-Boltzmann equation, we obtain the explicit forms of f_0 and f_1:

$$f_0 = f_N^{eq}, \tag{S11}$$

$$f_1 = f_R^{eq} - f_N^{eq} - \tau_N \left(\frac{\partial f_N^{eq}}{\partial t_1} + v_i \frac{\partial f_N^{eq}}{\partial x_{1i}} \right), \tag{S12}$$

and different order of approximations for the energy and heat-flux balance equations

$$\frac{\partial E}{\partial t_1} + \frac{\partial}{\partial x_{1i}} q_{0i} = 0, \tag{S13}$$

$$\frac{\partial q_{0i}}{\partial t_1} + \frac{\kappa_{0ji}}{\tau_R} \frac{\partial T}{\partial x_{1j}} = -\frac{1}{\tau_N} q_{0i} - \frac{1}{\tau_N} q_{1i}. \tag{S14}$$

To derive the hydrodynamic equations, we need to write the macroscopic variables of different orders using f_0 and f_1.

2.1 The zeroth-order result

The zeroth-order results are obtained by making the following approximation

$$f_0 = f_N^{eq} = f_R^{eq} + f_{N1}^{eq} \approx f_R^{eq} + \beta_B f_R^{eq} (f_R^{eq} + 1) \hbar \boldsymbol{k} \cdot \boldsymbol{u}. \tag{S15}$$

This approximation is valid if $|\hbar \boldsymbol{k} \cdot \boldsymbol{u}| \ll \hbar \omega$. It has been checked numerically[?].

2.1.1 Energy density

We consider the phonon energy density first. According to energy conservation, we know that $E = E_0$.

$$E = \sum_s \int \hbar \omega_{s\boldsymbol{k}} f_{N,s\boldsymbol{k}}^{eq} \frac{d\boldsymbol{k}}{(2\pi)^2} = \sum_s \int \hbar \omega_{s\boldsymbol{k}} f_{R,s\boldsymbol{k}}^{eq} \frac{d\boldsymbol{k}}{(2\pi)^2} + \sum_s \int \hbar \omega_{s\boldsymbol{k}} f_{R,s\boldsymbol{k}}^{eq} \left(1 + f_{R,s\boldsymbol{k}}^{eq}\right) \frac{\hbar \boldsymbol{k} \cdot \boldsymbol{u}}{k_B T} \frac{d\boldsymbol{k}}{(2\pi)^2}. \tag{S16}$$

The second term in the above equation vanishes because it's an odd function of $\boldsymbol{k}$. Thus we arrive at:

$$\begin{aligned} E &= \sum_s \int \hbar \omega_{s\boldsymbol{k}} f_{R,s\boldsymbol{k}}^{eq} \frac{d\boldsymbol{k}}{(2\pi)^2} \\ &= 2 \int \hbar v_g k \frac{1}{\exp(\frac{\hbar v_g k}{k_B T}) - 1} \frac{d\boldsymbol{k}}{(2\pi)^2} + \int \hbar a k^2 \frac{1}{\exp(\frac{\hbar a k^2}{k_B T}) - 1} \frac{d\boldsymbol{k}}{(2\pi)^2} \end{aligned} \tag{S17}$$

Writing it as summation of the linear E_L and quadratic E_N contributions, we get

$$E \equiv E_L + E_N, \tag{S18}$$

with

$$E_L = \frac{2}{\pi} \frac{(k_B T)^3}{(\hbar v_g)^2} Z(3), \quad E_N = \frac{\pi}{24} \frac{(k_B T)^2}{\hbar a}. \tag{S19}$$

Note that we have included the factor 2 to account the degeneracy of the linear phonon modes. From the energy above, we obtain the specific heat capacity as

$$C_L = \frac{6}{\pi} \frac{k_B^3 T^2}{(\hbar v_g)^2} Z(3), \quad C_N = \frac{\pi}{12} \frac{k_B^2 T}{\hbar a}. \tag{S20}$$

We also define an effective average energy and specific heat capacity as

$$\bar{E} = \frac{3}{2} E_L + 2 E_N, \quad \bar{C} = \frac{3}{2} C_L + 2 C_N, \tag{S21}$$

where $\bar{E}$ will be used in the expression for the heat flux.

2.1.2 Momentum density

Analogously, due to the momentum conservation of N-process, we have

$$\boldsymbol{p} = \boldsymbol{p}_0 = \sum_s \int \hbar \boldsymbol{k} f^{eq}_{N,s\boldsymbol{k}} \frac{d\boldsymbol{k}}{(2\pi)^2} = 2\int \hbar \boldsymbol{k} \frac{1}{\exp(\frac{\hbar v_g k - \hbar \boldsymbol{k}\cdot\boldsymbol{u}}{k_B T}) - 1} \frac{d\boldsymbol{k}}{(2\pi)^2} + \int \hbar \boldsymbol{k} \frac{1}{\exp(\frac{\hbar a k^2 - \hbar \boldsymbol{k}\cdot\boldsymbol{u}}{k_B T}) - 1} \frac{d\boldsymbol{k}}{(2\pi)^2} = \boldsymbol{p}_L + \boldsymbol{p}_N, \tag{S22}$$

where the linear and quadratic terms take the following forms

$$\boldsymbol{p}_L = \frac{3}{\pi} \frac{(k_B T)^3}{\hbar^2 v_g^4} Z(3) \boldsymbol{u} = \frac{3}{2} \frac{E_L}{v_g^2} \boldsymbol{u}, \tag{S23}$$

$$\boldsymbol{p}_N = \frac{1}{8\pi} \frac{k_B T}{a^2} \left[-\ln\left(1 - \exp(\frac{\beta_B \hbar u^2}{4a})\right)\right] \boldsymbol{u}. \tag{S24}$$

As discussed in the main text, to the first order in $\boldsymbol{u}$, $\boldsymbol{p}_N$ diverges logarithmically. While for small but finite $\boldsymbol{u}$, $\boldsymbol{p}_N$ is finite. Thus, we need to go beyond linear order in $\boldsymbol{u}$ when calculating $\boldsymbol{p}_N$.

2.1.3 Derivation of Eq. (S24)

We start from

$$\boldsymbol{p}_N = \int \hbar \boldsymbol{k} \frac{1}{\exp(\frac{\hbar a k^2 - \hbar \boldsymbol{k}\cdot\boldsymbol{u}}{k_B T}) - 1} \frac{d\boldsymbol{k}}{(2\pi)^2}. \tag{S25}$$

For simplicity, we set the drift velocity $\boldsymbol{u}$ to be parallel to the x axis, so

$$\begin{aligned}
(p_N)_x &= \int \hbar k \cos\theta \frac{1}{\exp(\frac{\hbar a k^2 - \hbar k u \cos\theta}{k_B T}) - 1} \frac{d\boldsymbol{k}}{(2\pi)^2} \\
&= \frac{\hbar}{(2\pi)^2} \sum_{n=0}^{\infty} \int k^2 \exp\left(-\frac{\hbar a k^2}{k_B T}(n+1)\right) dk \int_0^{2\pi} \cos\theta \exp\left(\frac{\hbar k u \cos\theta}{k_B T}(n+1)\right) d\theta \\
&= \frac{\hbar}{(2\pi)^2} \sum_{n=0}^{\infty} \int k^2 \exp\left(-\frac{\hbar a k^2}{k_B T}(n+1)\right) \cdot 2\pi J_1\left(1, \frac{\hbar k u}{k_B T}(n+1)\right) dk \\
&= \frac{\hbar}{2\pi} \sum_{n=0}^{\infty} \frac{\frac{\hbar u}{k_B T}(n+1) \cdot \exp(\frac{\left[\frac{\hbar u}{k_B T}(n+1)\right]^2}{4 \frac{\hbar a}{k_B T}(n+1)})}{4\left[\frac{\hbar a}{k_B T}(n+1)\right]^2} \\
&= \frac{1}{8\pi} \frac{k_B T}{a^2} u \sum_{n=0}^{\infty} \frac{1}{n+1} \exp\left(\frac{\hbar u^2}{4 k_B T a}(n+1)\right) \\
&= \frac{1}{8\pi} \frac{k_B T}{a^2} u \left[-\ln\left(1 - \exp(\frac{\hbar u^2}{4 k_B T a})\right)\right],
\end{aligned}$$

and

$$(p_N)_y = 0.$$

Putting together, we get

$$\boldsymbol{p}_N = \frac{1}{8\pi} \frac{k_B T}{a^2} \left[-\ln\left(1 - \exp(\frac{\beta_B \hbar u^2}{4a})\right)\right] \boldsymbol{u}.$$

2.1.4 Heat flux

The 0th order heat flux is calculated as

$$\boldsymbol{q}_0 = \sum_s \int \boldsymbol{v}_{s\boldsymbol{k}} \hbar \omega_{s\boldsymbol{k}} f^{eq}_{N,s\boldsymbol{k}} \frac{d\boldsymbol{k}}{(2\pi)^2} = \sum_s \int \boldsymbol{v}_{s\boldsymbol{k}} \hbar \omega_{s\boldsymbol{k}} f^{eq}_{R,s\boldsymbol{k}} \frac{d\boldsymbol{k}}{(2\pi)^2} + \sum_s \int \boldsymbol{v}_{s\boldsymbol{k}} \hbar \omega_{s\boldsymbol{k}} f^{eq}_{N1,s\boldsymbol{k}} \frac{d\boldsymbol{k}}{(2\pi)^2}. \tag{S26}$$

Since at thermal equilibrium, the heat flux is zero. Only the second term contributes

$$\boldsymbol{q}_0 = \sum_s \int \boldsymbol{v}_{s\boldsymbol{k}} \hbar \omega_{s\boldsymbol{k}} f^{eq}_{N1,s\boldsymbol{k}} \frac{d\boldsymbol{k}}{(2\pi)^2} = \frac{3}{2} E_L \boldsymbol{u} + 2 E_N \boldsymbol{u} \equiv \boldsymbol{q}_{0L} + \boldsymbol{q}_{0N}, \tag{S27}$$

where $\boldsymbol{q}_{0L}$, $\boldsymbol{q}_{0N}$ are the 0th order contributions of the linear and quadratic modes to the heat flux. Using Eq. (S21), we have

$$\boldsymbol{q}_0 = \bar{E} \boldsymbol{u}.$$

2.1.5 Thermal conductivity

The thermal conductivity tensor is

$$\overleftrightarrow{\boldsymbol{\kappa}}_0=\tau_R\sum_s\int\hbar\omega_{s\boldsymbol{k}}\boldsymbol{v}_{s\boldsymbol{k}}\boldsymbol{v}_{s\boldsymbol{k}}\frac{\partial f^{eq}_{N,s\boldsymbol{k}}}{\partial T}\frac{d\boldsymbol{k}}{(2\pi)^2}=\tau_R\sum_s\int\hbar\omega_{s\boldsymbol{k}}\boldsymbol{v}_{s\boldsymbol{k}}\boldsymbol{v}_{s\boldsymbol{k}}\frac{\partial f^{eq}_{R,s\boldsymbol{k}}}{\partial T}\frac{d\boldsymbol{k}}{(2\pi)^2}+\tau_R\sum_s\int\hbar\omega_{s\boldsymbol{k}}\boldsymbol{v}_{s\boldsymbol{k}}\boldsymbol{v}_{s\boldsymbol{k}}\frac{\partial f^{eq}_{N1,s\boldsymbol{k}}}{\partial T}\frac{d\boldsymbol{k}}{(2\pi)^2}. \tag{S28}$$

Likewise, the second term in the above equation is an odd function of $\boldsymbol{k}$ and thus is zero. We then have

$$\overleftrightarrow{\boldsymbol{\kappa}}_0=\overleftrightarrow{\boldsymbol{\kappa}}_{0L}+\overleftrightarrow{\boldsymbol{\kappa}}_{0N}, \tag{S29}$$

$$\overleftrightarrow{\boldsymbol{\kappa}}_{0L}=\overleftrightarrow{\boldsymbol{\kappa}}_{0N}=\frac{1}{2}C_Lv_g^2\tau_R\overleftrightarrow{\mathbf{I}}. \tag{S30}$$

It is noted that, given a constant τ_R, the thermal conductivity contributed from a quadratic mode is two times larger than that contributed from a linear mode. Both of them are independent of the details of the dispersion.

2.2 The first-order result

Now, we calculate the 1st order terms using the expansion

$$f_1=f_R^{eq}-f_N^{eq}-\tau_N\left(\frac{\partial f_N^{eq}}{\partial t_1}+v_i\frac{\partial f_N^{eq}}{\partial x_{1i}}\right). \tag{S31}$$

Based on the chain rule, we have

$$\frac{\partial f_N^{eq}}{\partial t_1}=\frac{\partial f_N^{eq}}{\partial u_j}\frac{\partial u_j}{\partial t_1}+\frac{\partial f_N^{eq}}{\partial T}\frac{\partial T}{\partial t_1}, \tag{S32}$$

$$\frac{\partial f_N^{eq}}{\partial x_{1i}}=\frac{\partial f_N^{eq}}{\partial u_j}\frac{\partial u_j}{\partial x_{1i}}+\frac{\partial f_N^{eq}}{\partial T}\frac{\partial T}{\partial x_{1i}}. \tag{S33}$$

To proceed, we have two options for the derivation of G-K equation. These two options are exactly the same for phonons with linear dispersion, i.e. the Debye model. But we must pay attention to the quadratic phonon mode here, it leads to different results. The underline physics is that, for phonons with linear dispersion, the momentum density and the heat flux are proportional to each other

$$\boldsymbol{q}_L=v_g^2\boldsymbol{p}_L. \tag{S34}$$

The group velocity v_g is constant for phonons with linear dispersion. But for quadratic dispersion, this simple relationship does not hold, due to the wavevector dependence of v_g, i.e. $v_g\propto k$.

Here, to consider heat transport, we start from Eqs. (S13-S14) and arrive at a lengthy expression for f_1

$$\begin{aligned}f_1=&-\tau_Nf_R^{eq}\left(f_R^{eq}+1\right)\left\{\frac{\hbar k_i}{k_BT}\left(-\frac{2C_Lv_g^2}{3E_L+4E_N}\frac{\partial T}{\partial x_{1i}}+\frac{u_i}{3E_L+4E_N}\frac{3C_L+4C_N}{C}\right.\right.\\&\left.\cdot\frac{\partial q_{0j}}{\partial x_{1j}}-\frac{2}{3E_L+4E_N}\frac{q_{1i}}{\tau_N}\right)+\left(\frac{v}{k}k_i\frac{\partial T}{\partial x_{1i}}-\frac{1}{C}\frac{\partial q_{0j}}{\partial x_{1j}}\right)\left[\frac{\hbar\omega}{k_BT^2}+\right.\\&\left.\left.\frac{\hbar\omega}{k_BT^2}\left(2f_R^{eq}+1\right)\frac{\hbar\boldsymbol{k}\cdot\boldsymbol{u}}{k_BT}-\frac{\hbar\boldsymbol{k}\cdot\boldsymbol{u}}{k_BT^2}\right]+\frac{v}{k}\frac{\hbar k_ik_j}{k_BT}\frac{\partial}{\partial x_{1i}}\left(\frac{2q_{0j}}{3E_L+4E_N}\right)\right\}.\end{aligned} \tag{S35}$$

Note that, in the expression for f_1 we have one term $\propto q_{1i}$. On the other hand, to get q_{1i} we need to know f_1. This means the equations are coupled together and have to be solved self-consistently. Fortunately, the q_{1i} term in Eq. (S35) is odd in k and does not contribute to $\overleftrightarrow{\boldsymbol{\kappa}}_1$ and the viscosity coefficients in the final G-K equation. We get for $\overleftrightarrow{\boldsymbol{\kappa}}_1$

$$\overleftrightarrow{\boldsymbol{\kappa}}_1=\tau_R\left(\nabla\cdot\overleftrightarrow{\mathbf{Q}}\right)\frac{1}{\nabla T}, \tag{S36}$$

where

$$\overleftrightarrow{\mathbf{Q}} = \overleftrightarrow{\mathbf{Q}}_I + \overleftrightarrow{\mathbf{Q}}_{II} + \overleftrightarrow{\mathbf{Q}}_{III}, \tag{S37}$$

$$(Q_I)_{mn} = \tau_N v_g^2 \frac{C_L}{C} \frac{\partial q_{0j}}{\partial x_{1j}} \delta_{mn}, \tag{S38}$$

$$(Q_{II})_{mn} = -\frac{9}{8} v_g^2 E_L \tau_N \left[\frac{\partial}{\partial x_{1i}} \left(\frac{q_{0i}}{\bar{E}} \right) \delta_{mn} + \frac{\partial}{\partial x_{1m}} \left(\frac{q_{0n}}{\bar{E}} \right) + \frac{\partial}{\partial x_{1n}} \left(\frac{q_{0m}}{\bar{E}} \right) \right], \tag{S39}$$

$$(Q_{III})_{mn} = -\frac{9}{4} \frac{v_g^2 \tau_N}{T} \left[(q_{0L})_i \frac{\partial T}{\partial x_{1i}} \delta_{mn} + (q_{0L})_n \frac{\partial T}{\partial x_{1m}} + (q_{0L})_m \frac{\partial T}{\partial x_{1n}} \right]. \tag{S40}$$

2.3 The heat G-K equation

Substituting the calculated 0th and 1st order results into

$$\frac{\partial \boldsymbol{q}}{\partial t} + \frac{\overleftrightarrow{\kappa}}{\tau_R} \cdot \nabla T = -\frac{\boldsymbol{q}}{\tau_R} - \frac{\boldsymbol{q} - \boldsymbol{q}_0}{\tau_N}, \tag{S41}$$

neglecting the nonlinear effects resulting from the product of heat flux and temperature gradient in κ_1 and keeping only the lowest order term in the heat flux $\boldsymbol{q} \approx \boldsymbol{q}_0$, we get the G-K equation for heat flux

$$\frac{\partial \boldsymbol{q}}{\partial t} + \frac{\kappa_0}{\tau_R} \nabla T + \frac{1}{\tau_R} \boldsymbol{q} = \eta \left[\nabla^2 \boldsymbol{q} + 2\nabla(\nabla \cdot \boldsymbol{q}) \right] - \zeta \nabla(\nabla \cdot \boldsymbol{q}). \tag{S42}$$

The transport coefficients are $\kappa_0 = \alpha C v_g^2 \tau_R$, $\eta = \beta v_g^2 \tau_N$, $\zeta = \alpha v_g^2 \tau_N$, with $\alpha = C_L/C$, $\beta = 9E_L/8\bar{E}$, respectively. All the three transport coefficients can be divided into contributions from the linear and quadratic phonon modes, respectively. Denoting them with subscripts L and N, we have $\kappa_{0L} = \kappa_{0N} = C_L v_g^2 \tau_R/2$, $\eta_L = \eta_N/2 = 3E_L/(8\bar{E}) v_g^2 \tau_N$, $\zeta_L = \zeta_N = C_L/(2C) v_g^2 \tau_N$. It can be checked that, our results reduce to that of Debye model if we ignore the quadratic phonon mode.

3 Steady state heat flow in a ribbon

We proceed to analyse the hydrodynamic heat transport in a graphene nano-ribbon. At steady state, we have $\partial \boldsymbol{q}/\partial t = \partial E/\partial t = 0$ and Eq. (S42) reduce to

$$\eta \nabla^2 \boldsymbol{q} - \tau_R^{-1} \boldsymbol{q} = C_L v_g^2 \nabla T. \tag{S43}$$

Similar to Ref.[?], we introduce a stream function ψ with $\boldsymbol{q} = \boldsymbol{z} \times \nabla \psi$. A flow of heat current from a point source $I(x) = I\delta(x)$ is injected into the ribbon and collected at the opposite side. So the normal heat flux is

$$q_y(x,y)_{y=0,w} = I(x). \tag{S44}$$

With non-slip boundary condition, we have

$$q_x(x,0) = q_x(x,w) = 0, \tag{S45}$$

w is the width of the ribbon. With the boundary conditions above, we can get

$$\psi_k(y) = -\frac{I}{ik} \sum_{\pm} \left[a_\pm \exp(\pm ky) + b_\pm \exp(\pm k' y) \right], \quad \psi(x,y) = \frac{1}{2\pi} \int dk e^{ikx} \psi_k(y), \tag{S46}$$

$$T(x,y) = \frac{I}{\kappa_0 2\pi} \int_{-\infty}^{\infty} e^{ikx} \left[a_+(k) e^{ky} - a_-(k) e^{-ky} \right] \frac{dk}{k}, \tag{S47}$$

with $k'^2 = k^2 + \frac{1/\tau_R}{\eta}$ and

$$a_+ = \frac{\left(e^{k'w} - 1\right) k'}{(k - k')\left(1 - e^{(k+k')w}\right) + (k + k')\left(e^{k'w} - e^{kw}\right)},$$
$$a_- = \frac{e^{kw}\left(e^{k'w} - 1\right) k'}{(k - k')\left(1 - e^{(k+k')w}\right) + (k + k')\left(e^{k'w} - e^{kw}\right)},$$
$$b_+ = \frac{\left(e^{kw} - 1\right) k}{(k' - k)\left(1 - e^{(k+k')w}\right) + (k + k')\left(e^{kw} - e^{k'w}\right)},$$
$$b_- = \frac{\left(e^{kw} - 1\right) k e^{k'w}}{(k' - k)\left(1 - e^{(k+k')w}\right) + (k + k')\left(e^{kw} - e^{k'w}\right)}.$$

References

1. Guo, Y. & Wang, M. Phonon hydrodynamics and its applications in nanoscale heat transport. *Phys. Rep.* **595**, 1 – 44, DOI: https://doi.org/10.1016/j.physrep.2015.07.003 (2015).

2. Lee, S., Broido, D., Esfarjani, K. & Chen, G. Hydrodynamic phonon transport in suspended graphene. *Nat. Commun.* **6**, 6290, DOI: 10.1038/ncomms7290 (2015).

3. Levitov, L. & Falkovich, G. Electron viscosity, current vortices and negative nonlocal resistance in graphene. *Nat. Phys.* **12**, 672–676, DOI: 10.1038/nphys3667 (2016).
